# Identification of a unique allele in the quantitative trait locus for crown root number in *japonica* rice from Japan using genome-wide association studies

**DOI:** 10.1270/jsbbs.22010

**Published:** 2022-07-01

**Authors:** Shota Teramoto, Masanori Yamasaki, Yusaku Uga

**Affiliations:** 1 Institute of Crop Science, National Agriculture and Food Research Organization, Tsukuba, Ibaraki 305-8518, Japan; 2 Food Resources Education and Research Center, Graduate School of Agricultural Science, Kobe University, Kasai, Hyogo 675-2103, Japan

**Keywords:** genome-wide association studies, quantitative trait loci, adventitious root, nodal root, fibrous root

## Abstract

To explore the genetic resources that could be utilized to help improve root system architecture phenotypes in rice (*Oryza sativa*), we have conducted genome-wide association studies to investigate maximum root length and crown root number in 135 10-day-old Japanese rice accessions grown hydroponically. We identified a quantitative trait locus for crown root number at approximately 32.7 Mbp on chromosome 4 and designated it *qNCR1* (*quantitative trait locus for Number of Crown Root 1*). A linkage disequilibrium map around *qNCR1* suggested that three candidate genes are involved in crown root number: a cullin (*LOC_Os04g55030*), a gibberellin 20 oxidase 8 (*LOC_Os04g55070*), and a cyclic nucleotide-gated ion channel (*LOC_Os04g55080*). The combination of haplotypes for each gene was designated as a haploblock, and haploblocks 1, 2, and 3 were defined. Compared to haploblock 1, the accessions with haploblocks 2 and 3 had fewer crown roots; approximately 5% and 10% reductions in 10-day-old plants and 15% and 25% reductions in 42-day-old plants, respectively. A Japanese leading variety Koshihikari and its progenies harbored haploblock 3. Their crown root number could potentially be improved using haploblocks 1 and 2.

## Introduction

Root system architecture (RSA) results from the deployment of various roots in the soil (Lynch 1995). In general, RSA can either help or limit the development of roots in the soil, which respectively, can positively or negatively affect plant growth (Gowariker *et al.* 2009, Lynch 1995). Rice (*Oryza sativa*) is one of the top three grain crops in the world, and several studies have shown that superior rice RSAs can result in increased yields in stressed soils. For example, rice productivity could be maintained under drought conditions if the depth of the root distribution was increased (Uga *et al.* 2013). While exposing the roots to the soil surface led to increased rice yields in saline paddies (Kitomi *et al.* 2020). Therefore, we assumed that there is an optimal rice RSA for each soil environment. The major challenge in RSA breeding is to develop new cultivars which can be adapted to different environments by improving RSAs using genetic resources (Uga 2021, Uga *et al.* 2015a).

Rice plants have fibrous root systems which consist of adventitious and lateral roots (Osmont *et al.* 2007), which are determined by three major components: root length, number, and angle. Root length, especially adventitious root length, affects the size of the RSA. An increase in maximum root length is related to the acquisition of water and nutrients (Courtois *et al.* 2009), and consequently, maximum root length is a target trait when breeding crops to efficiently use water and nutrient resources in the soil. Root number does not affect RSA size, but it does affect the root density. The higher the root density, the faster the absorption of water and nutrients (Marschner and Rengel 2012); which is further evidence by that fact in rice, root density and grain yield were found to be positively correlated (Yang *et al.* 2012). Therefore, root number is also a target when breeding to improve RSA. Root angle is the elongation angle of the roots, and is especially used to assess the adventitious roots in monocotyledons (Oyanagi 1994, Oyanagi *et al.* 1993, Trachsel *et al.* 2013, Uga *et al.* 2011). Changes in the root angle have a significant impact on the RSA as they affect the root distribution in the soil (Ge *et al.* 2000). Therefore, root angle modifications are widely targeted to improve the RSA in rice (Kitomi *et al.* 2020, Uga *et al.* 2013). Taken together, the combination of these three components should be targeted to improve RSA in rice.

Many quantitative trait loci (QTLs) responsible for RSA-related traits have been identified in rice. Previously, QTLs associated with root length (Courtois *et al.* 2009, Kitomi *et al.* 2018, Obara *et al.* 2014, Steele *et al.* 2006, Zhang *et al.* 2001), root number (Ali *et al.* 2000, Courtois *et al.* 2009, Islam *et al.* 2021, Ray *et al.* 1996), and root angle (Uga *et al.* 2011, 2013, 2015b) have all been identified. However, except for two genes associated with root angle (Kitomi *et al.* 2020, Uga *et al.* 2013), the genes responsible for most QTLs have not been isolated. Thus, the isolation of QTLs associated with root length and number is also required to further improve rice RSA.

Genome-wide association studies (GWAS) are powerful tools that can be utilized to identify the genes associated with agronomic traits; and the associations of nucleotide polymorphisms and phenotypic variances are best analyzed using a diverse population (Yano *et al.* 2016). Indeed, some RSA-related QTLs have previously been detected using GWAS in wheat and rice (Alemu *et al.* 2021, Beyer *et al.* 2019, Wang *et al.* 2019a), however, most were identified using bi-parental populations. GWAS generally narrow down the candidate genes inside linkage disequilibrium (LD) blocks. As the average size of LD blocks in cultivated rice is <500 kbp (Mather *et al.* 2007), we theorized that candidate genes in QTLs related to root length and number could be identified using GWAS.

In this study, using a GWAS mapping population that consisted of temperate *japonica* rice cultivars (Chigira *et al.* 2020), we performed GWAS to identify the QTL(s) associated with root length and number and evaluated the effects of the detected QTL(s) on phenotype. Furthermore, we investigated the geographic proportions in the different haploblocks and have discussed their potential applications.

## Materials and Methods

### Plant materials and cultivation

In this investigation 135 temperate *japonica* rice accessions, which were previously cultivated or used as parental breeding in Japan, were utilized (Chigira *et al.* 2020). As the population structure was low but there was a large phenotypic diversity, the accessions were suitable for a GWAS. Information on the accessions used, such as cultivar name and origin, was previously reported (Chigira *et al.* 2020).

To investigate maximum root length, crown root number, and shoot length at the early seedling stage of development, hydroponic cultivation with floating nets was performed. Seeds were surface sterilized with tap water containing fungicide at 15°C and immersed in tap water at 30°C for 2 d. The imbibed seeds were transferred onto a net floating on a 1/4-strength hydroponic solution based on Kimura B solution (Kitomi *et al.* 2018, Yoshida *et al.* 1976). The composition of the hydroponic solution was as follows: 91.25 μM (NH_4_)_2_SO_4_, 22.75 μM K_2_SO_4_, 136.75 μM MgSO_4_, 45.75 μM KNO_3_, 91.25 μM Ca(NO_3_)_2_, 45.5 μM KH_2_PO_4_, and 4.45 μM FeC_6_H_5_O_7_. The pH was adjusted to 5.5 with 5 mM MES (C_6_H_13_NO_4_S), HCl, and KOH. The hydroponic solution was changed every 3 or 4 d. Room temperature was defined as 30°C for 12 h during the day and 26°C for 12 h during the night in a greenhouse environment. Irradiation using 400 W metal-halide lamps was provided during the day. Poorly growing plants were thinned out prior to measurements. The seedlings were cultivated for 10 d in the greenhouse, and then their maximum root length, crown root number, and shoot length were measured. Data were collected from a maximum of ten individuals from each accession. Two cultivation trials were conducted.

To investigate crown root number in the late seedling stage of development, hydroponic cultivation with stainless-steel mesh baskets was performed. The mesh baskets (7.5 cm diameter × 5.0 cm depth) were filled with nutrient-poor soil Akadama (Ikubyo-Shibaue-Yodo, Shidara, Kanuma, Tochigi, Japan). Then, a germinated seed was sown at the center of each basket. Water and nutrients were supplied using a hydroponic solution: the 1/4- and 1/2-strength hydroponic solution, in which the pH was adjusted to 5.8 with HCl and KOH. The hydroponic solution was changed every 3 or 4 days. Room temperature and irradiation conditions were as defined previously for the maximum root length experiment. The seedlings were cultivated for six weeks in the greenhouse, after which their crown root numbers were counted. Data were collected from three individuals of each cultivar. Two cultivation trials were conducted.

### Genome-wide association analysis

GWAS and gene-based association studies were performed according to a previous study (Chigira *et al.* 2020): we used a total of 670,069 SNPs and IndDels, and the haplotypes of 14,274 rice genes among the 135 temperate *japonica* rice cultivars. The linear mixed model incorporated the kinship data (Yu *et al.* 2006). A LD map was drawn using the “LDheatmap” R package (Shin *et al.* 2006) with the sequence variant data from the 135 accessions. The LD block was used to determine the candidate region.

### Detection of the candidate genes

The criteria for the candidate genes was the same as the previous study (Chigira *et al.* 2020); the genes in the candidate region whose *p* value in the gene-based association study was <0.0001 in both the first and second trials were identified as candidate genes. A set of gene IDs and descriptions was downloaded from the Rice Annotation Project Database (Kawahara *et al.* 2013, Sakai *et al.* 2013).

### Sequence logo analysis

Genomic sequences of a homologous gene in rice, Arabidopsis (*Arabidopsis thalianla*), *Cyanidioschyzon merolae*, and *Saccharomyces cerevisiae* were collected from the SALDA database version 3.0 (Mihara *et al.* 2009). We extracted dozens of amino acid sequences around the single nucleotide polymorphism position, and created a sequence logo using the “ggseqlogo” R package (Wagih 2017).

### Analysis of geographical distribution of haplotypes

Origins of the accessions were grouped into nine region categories, which were all within Japan: Hokkaido, Tohoku, Kanto, Hokuriku, Tokai, Kinki, Shikoku, Chugoku, and Kyushu. The percentage of haplotypes in each category was drawn on a Japanese map using the “scatterpie” R package. The map was drawn using the “sf” R package (Pebesma 2018) with a shape file downloaded from “https://www.naturalearthdata.com/”.

## Results

### Phenotypic variations in the root traits of Japanese rice accessions

**Fig. 1.**
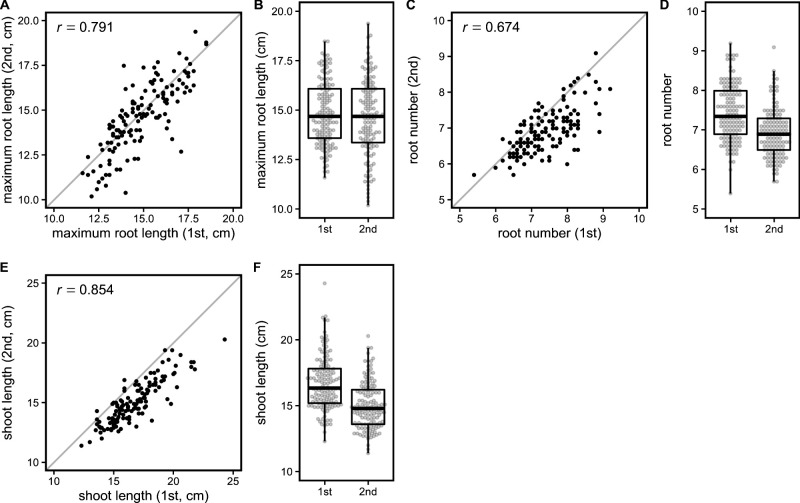
Phenotypic variations in Japanese rice accessions. Maximum root length (A and B), root number (C and D), and shoot length (E and F). Scatter plots of 1st and 2nd trials (A, C, and E). Pearson’s correlation coefficient is shown in the upper left corner of the scatter plot. The straight line with a slope of 1 and intercept of 0 were drawn. Boxplots of 1st and 2nd trials (B, D, and F). The top and bottom of each box indicates the first and third quartiles, respectively. The center line indicates the median, and the whiskers show the range of the observed values within 1.5 times the interquartile range from the hinges. The constituents of the box plot were marked as beeswarm points.

We cultivated 135 Japanese rice accessions hydroponically for 10 d after sowing to determine their maximum root length, root number, and shoot length (Fig. 1). For the maximum root length, a positive correlation between the first and second trials was observed (Fig. 1A) and there were no differences in the median values between the trials (Fig. 1B). This indicated that the differences in the first and second trials did not affect the maximum root length. For root number, a positive correlation between the first and second trials was observed (Fig. 1C), and the root number was slightly larger in the first trial (Fig. 1D). A similar tendency was observed in the results for shoot length (Fig. 1E, 1F), indicating that the first trial had a more positive effect on growth than the second. As positive correlation was observed between the three measured traits (Fig. 1A, 1C, 1E), we assumed that environmental differences were small in this study, and that the variations in the phenotypic data would reflect the genetic diversity of the population.

### Candidate gene for root traits

**Fig. 2.**
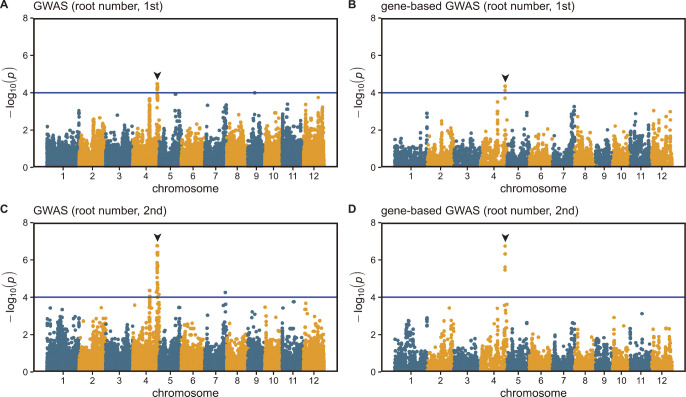
Manhattan plots for root number. GWAS (A) and gene-based GWAS results (B) of the 1st trial. GWAS (C) and gene-based GWAS results (D) of the 2nd trial. The horizontal blue line indicates the threshold of the *p* value 0.0001. Arrowheads indicate the QTL commonly detected in A, B, C, and D.

To identify the candidate genes affecting the root traits, a GWAS was conducted using the phenotypic data from the 135 rice cultivars. Manhattan plots for the maximum root length (Supplemental Fig. 1), root number (Fig. 2), and shoot length (Supplemental Fig. 2) are shown. We obtained one significant peak around 32.7 Mbp on chromosome 4 for root number (Fig. 2) and two significant peaks around 34.9 Mbp on chromosome 3 and 25.4 Mbp on chromosome 8 for shoot length (Supplemental Fig. 2) but none for maximum root length (Supplemental Fig. 1). The loci for the significant peaks associated with the shoot length and root number traits were different, which indicates that the candidate genes for root number and root length were not the same. The QTL for root number was named *qNCR1* (*quantitative trait locus for Number of Crown Root 1*).

**Fig. 3.**
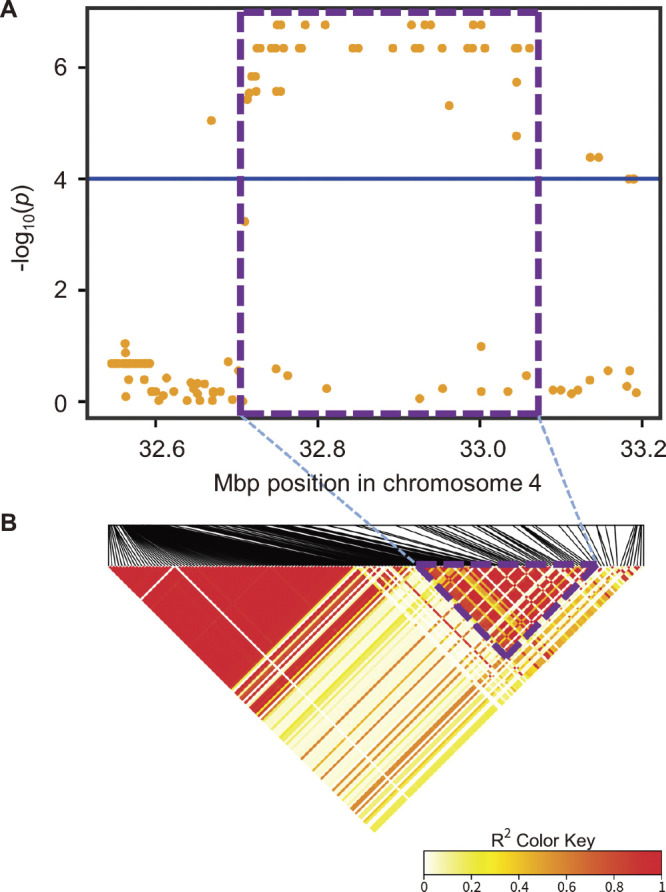
Mapping the candidate genes associated with root number. (A) An enlarged view of the Manhattan plot for the GWAS for root number of the 2nd trial (Fig. 2c) and (B) the corresponding LD plot. The horizontal blue line indicates the threshold of the *p* value 0.0001. The area enclosed by the purple dashed line indicates the candidate region.

To identify the candidate genes affecting root number, we focused the Manhattan plot and LD map around *qNCR1* (Fig. 3). Based on the LD heatmap (Fig. 3B), we delimited approximately 334-kbp candidate region (Fig. 3A). Genes with a *p* value <0.0001 in the gene-based GWAS are considered candidate genes, and this region had nine candidate genes [Table 1 (Ci *et al.* 2021, Gingerich *et al.* 2005, Nawaz *et al.* 2014)]. Excluding transposon-related genes, there were four candidate genes, namely *LOC_Os04g55030*, *LOC_Os04g55070*, *LOC_Os04g55080*, and *LOC_Os04g55130*. We investigated the expression profile of them by RiceXPro database (Sato *et al.* 2011a, 2011b, 2013) and found that *LOC_Os04g55030* and *LOC_Os04g55080* were expressed ubiquitously in all tissues including roots and *LOC_Os04g55070* and *LOC_Os04g55130* were highly expressed in roots and inflorescence, respectively (Supplemental Figs. 3–6). Focusing on genes that are highly expressed in the roots, three annotated genes were left as candidates: a cullin (*LOC_Os04g55030*), a gibberellin (GA) 20 oxidase (GA20ox, *LOC_Os04g55070*), and a cyclic nucleotide-gated ion channel (*CNGC*, *LOC_Os04g55080*). All three candidates had missense mutations in the GWAS population (Supplemental Fig. 7). Among them, *LOC_Os04g55030* and *LOC_Os04g55070* showed a change in the charge of the amino acid (Supplemental Fig. 7A, 7B). Sequence logo analysis revealed that the 223rd amino acid of *LOC_Os04g55030* was highly conserved as an amino acid with a negative charge among the 51 homologous sequences (Supplemental Fig. 8), suggesting that a change from a negative to a positive charge, would influence its function. However, as any gene in the LD block could be a candidate gene, we could not indicate the causal gene for root number.

Based on the variant call results in the coding sequences, we detected two haplotypes in *LOC_Os04g55030*, *LOC_Os04g55070*, and *LOC_Os04g55080* (Supplemental Fig. 7); haplotype 1, which is identical to the RAP-DB sequence (Kawahara *et al.* 2013, Sakai *et al.* 2013), and haplotype 2. Haplotypes of *LOC_Os04g55030* and *LOC_Os04g55080* were completely linked. To investigate the influence of haplotypes 1 and 2 on root number, we compared the root numbers from the 135 rice cultivars 10 d (Supplemental Fig. 9A, 9B) and 42 d after sowing (Supplemental Fig. 9C, 9D). The group harboring haplotype 2 had significantly lower root numbers when compared to the group harboring haplotype 1 in all conditions (Supplemental Fig. 9). The reduction in the number of roots was approximately 10% 10 d and 25% 42 d after sowing (Supplemental Fig. 9).

**Fig. 4.**
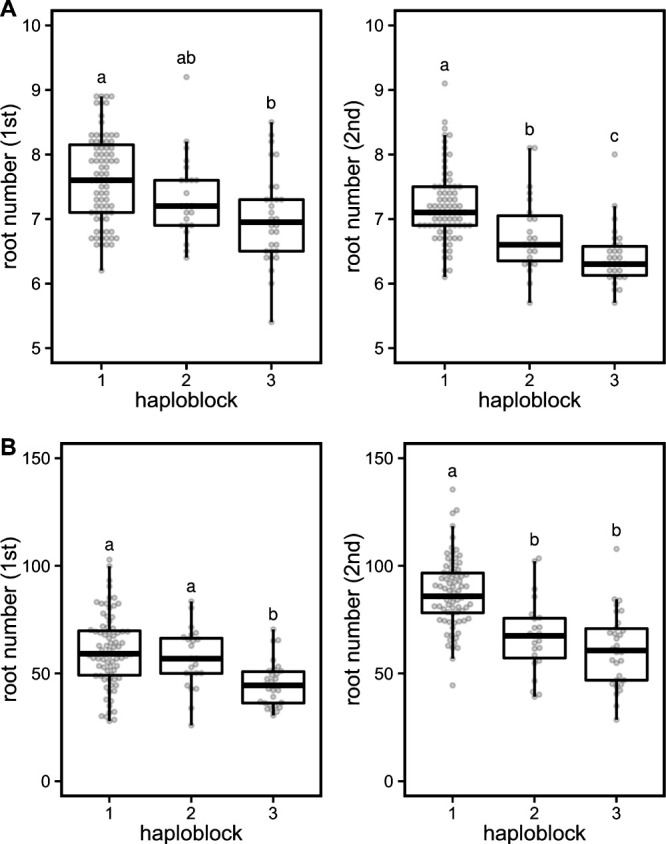
The effects of haploblocks on crown root number. Boxplots for the root numbers in haploblocks 1, 2 and 3 at (A) early growth and (B) middle growth stages. The first and second trials are shown. The top and bottom of the boxes indicates the first and third quartiles, respectively. The center line indicates the median, and the whiskers show the range of observed values within 1.5 times the interquartile range from the hinges. The constituents of the box plot were marked as beeswarm points. Differences between the haplotypes were analyzed using the Steel-Dwass test.

The combination of the haplotypes for each gene were designated as a haploblock, and haploblocks 1, 2, and 3 were defined. Haploblock 1 harbored haplotype 1 at all genes. Haploblock 2 harbored haplotype 1 at *LOC_Os04g55030* and *LOC_Os04g55080* and haplotype 2 at *LOC_Os04g55070*. Haploblock 3 harbored haplotype 2 at all genes. When compared to haploblock 1, the accessions with haploblocks 2 and 3 had fewer crown roots, with approximately 5% and 10% reductions in the 10 d old plants and 15% and 25% reductions in the 42 d old plants, respectively (Fig. 4). Haploblock 3 reproducibly reduced the crown root number, while haploblock 2 did not (Fig. 4).

### Distribution of the three haploblocks

**Fig. 5.**
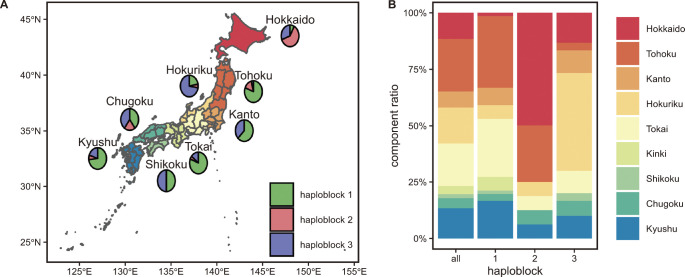
Distribution of the haploblocks in Japan. (A) Geographic distribution of haploblocks 1, 2, and 3 in Japan. Japan is divided into nine subsectors (namely the Hokkaido, Tohoku, Kanto, Hokuriku, Tokai, Kinki, Shikoku, Chugoku, and Kyushu regions), and the percentage of haploblocks 1, 2, and 3 in each subsector is shown. (B) The percentage of each haploblock in each subsector is shown.

To investigate the distribution of the three haploblocks, we obtained genomic sequences of 69 accessions from the world rice core collection (WRC) and 50 accessions from the rice core collection of Japanese landraces [JRC; https://ricegenome-corecollection.dna.affrc.go.jp/) (Tanaka *et al.* 2020, 2021)]. Haploblock 2 was distributed among almost all world-wide accessions whereas haploblocks 1 and 3 were mainly distributed in the Japanese accessions (Supplemental Table 1). A Japanese leading variety Koshihikari (Kobayashi *et al.* 2018) and its progenies harbored haploblock 3, implying that haploblock 3 was spread throughout Japan during breeding processes that utilized Koshihikari. We investigated a more detailed study of the distribution of the three haploblocks in Japan using the rice accession sequences that had been used in the GWAS (Fig. 5). We found that haploblocks 2 and 3 were concentrated in the Hokkaido and Hokuriku regions in Japan, respectively (Fig. 5). Hokkaido is the northernmost region of Japan and is one of the northern limits for rice cultivation (Fujino *et al.* 2019). Hokuriku is the region where the Koshihikari cultivar was first developed (Kobayashi *et al.* 2018).

## Discussion

This study detected a QTL for crown root number, *qNCR1*, for which three possible candidate genes were also identified, a cullin (*LOC_Os04g55030*), a GA20ox (*LOC_Os04g55070*), and a CNGC (*LOC_Os04g55080*), although any gene in the LD block could be a candidate. The cullin protein assembles multi-subunit Cullin-RING E3 ubiquitin ligase complexes, involved in various functions, such as cell-cycle control, DNA replication, and development (Sarikas *et al.* 2011). GA20ox is a GA biosynthesis enzyme, involved in many aspects of plant growth and development (Spielmeyer *et al.* 2002). CNGC is a nonselective cation channel (Kaupp and Seifert 2002). We defined three haploblocks based on their combinations of the haplotypes for *LOC_Os04g55030*, *LOC_Os04g55070*, and *LOC_Os04g55080*. Accessions harboring haploblocks 1 and 3 had the highest and the lowest crown root numbers, respectively (Fig. 4). The root numbers in accessions with haploblocks 2 and 3, when compared to those with haploblock 1, were approximately 5% and 10% lower in the 10 d old plants and 15% and 25% lower in the 42 d old plants, respectively. This indicated that the root number could be improved exchanging these haploblocks in *qNCR1*. Because haploblock 1 harbored haplotype 1 at all genes, and haploblock 2 harbored haplotype 1 at *LOC_Os04g55030* and *LOC_Os04g55080* and haplotype 2 at *LOC_Os04g55070*, at least *LOC_Os04g55070* might affect the crown root number. Furthermore, haploblock 3 harbored haplotype 2 at all genes, indicating that *LOC_Os04g55030* and/or *LOC_Os04g55080* might affect the crown root number additively with *LOC_Os04g55070*. Therefore, we assumed that at least two genes associated with the crown root number.

*LOC_Os04g55030*, is a *CULLIN3* (*CUL3*) gene family member, *OsCUL3b* (Gingerich *et al.* 2005). CUL3 is a component of CUL3 E3 ligases, and it binds target substrates via “Bric a brac, Tramtrack and Broad Complex/Pox virus, and Zinc finger” (BTB/POZ) proteins and it binds a ubiquitin conjugating enzyme, E2 ligase, resulting in the ubiquitination of a target sequence (Ban and Estelle 2021). As 80 and 149 BTB-domain proteins are present in the Arabidopsis and rice genomes, respectively, and the majority of them interact with CUL3 (Ban and Estelle 2021), these complexes may patriciate in various regulations of plant development. Arabidopsis genomes contain two *CUL3* genes, *AtCUL3a* and *AtCUL3b*. It was reported that mutations in both *AtCUL3a* and *AtCUL3b* resulted in embryo development arrest (Figueroa *et al.* 2005) and that AtCUL3a is involved in regulating immunity by degrading AtNPR1, a homolog of OsNPR1 (Spoel *et al.* 2009). These results indicated that CUL3s are involved in essential development processes and immune responses. Furthermore, AtCUL3a interacts with ETO1, a BTB-domain protein which directly interacts with the rate-limiting enzyme in ethylene biosynthesis 1-aminocyclopropane-1-carboxylate synthase 5 (ACS5), to degrade ACS5 (Wang *et al.* 2004). A CUL3 hypomorphic mutant failed to degrade ACS5 and exhibits a constitutive ethylene response (Thomann *et al.* 2009). The rice genome contains three *CUL3* genes, *OsCUL3a*, *OsCUL3b*, and *OsCUL3c* (Gingerich *et al.* 2005), of which the analysis of *OsCUL3a*, which is involved in cell death and immune responses, is the most advanced (Gao *et al.* 2020, Liu *et al.* 2017). It is reported that mutations in *OsCUL3a* result in a severe cell death phenotype at the tillering stage and enhanced resistance to pathogens by degrading OsNPR1, a positive regulator of cell death in rice, via the 26S proteasome (Liu *et al.* 2017). There is no previous evidence that OsCUL3b influences crown root number, but we considered that *OsCUL3b* could be involved with crown root number as adequate auxin signaling is important for crown root emergence and CUL3 potentially interacts with BTB-domain proteins, some of which reportedly participate in auxin-mediated plant development (Cheng *et al.* 2007, Mandadi *et al.* 2009).

*LOC_Os04g55070* is *OsGA20ox8*, an enzyme that is involved in bioactive GAs synthesis (Ci *et al.* 2021). The rice genome contains eight *OsGA20ox* genes, *OsGA20ox1–8* (Ci *et al.* 2021, Han and Zhu 2011). The most well-known *GA20ox* gene is *GA20ox2*, as its loss-of-function allele was used in the green revolution (Ashikari *et al.* 2002). *GA20ox4* is involved in panicle length, as higher expression levels of *GA20ox4* in young panicles results in panicle rachis elongation (Agata *et al.* 2020). OsGA20ox1 (Oikawa *et al.* 2004) and OsGA20ox3 (Qin *et al.* 2013) are also involved in GAs synthesis. OsGA20ox6 was reported to be a dioxygenase that converts indole-3-acetic acid (IAA) into inactive OxIAA (Zhao *et al.* 2013), indicating that not all OsGA20oxs are involved in GA metabolism (Liu *et al.* 2020). Another example is OsGA20ox7, which is involved in salicylic acid homeostasis (Liu *et al.* 2020). As mentioned above, GAs are involved in many aspects of plant growth and development (Spielmeyer *et al.* 2002), and changes in their levels also influence adventitious root formation (Lo *et al.* 2008, Mauriat *et al.* 2014). Although the roles of GA20ox8 (*LOC_Os04g55070*) are currently unclear, GA20ox8 may influence crown root number.

*LOC_Os04g55080* is *OsCNGC8* (Nawaz *et al.* 2014), which is a member of *CNGC* encoding a nonselective cation channel permeable to cations such as K^+^, Na^+^, and Ca^2+^ (Kaupp and Seifert 2002). The rice genome contains sixteen *OsCNGC* genes, *OsCNGC1–16* (Nawaz *et al.* 2014). *OsCNGC1* contributed to salt stress tolerance as they can help to avert toxic Na^+^ influxes (Assaha *et al.* 2017). *OsCNGC13* is permeable to Ca^2+^ to facilitate pollen tube growth (Xu *et al.* 2017). *OsCNGC9* plays an important role in the resistance to rice blast disease by mediating pathogen-associated molecular pattern (PAMP)-induced Ca^2+^ influxes (Wang *et al.* 2019b). *OsCNGC14* and *OsCNGC16* are involved in the tolerance to heat and chilling stresses and their loss-of -function can reduce or abolish cytosolic calcium signals that are induced by either heat or chilling stresses (Cui *et al.* 2020). As shown above, the CNGC family has a wide range of functions, from simple ion transport to the regulation of calcium signaling. It is possible that there are functions in the CNGC family that affect root number, but the specific functions of *OsCNGC8* are still unknown.

Taken together, all three candidate genes, *LOC_Os04g55030*, *LOC_Os04g55070*, and *LOC_Os04g55080*, are potentially causal gene(s) for *qNCR1*. To confirm the identity of the genes involved, it will be necessary to make knockout mutants by CRISPR/Cas9 system and/or conduct complementation tests in the future. It seems, however, that it may be possible to improve root systems by using haploblocks of *qNCR1* even though the causal genes were not confirmed in this study. When compared to haploblock 1, haploblocks 2 and 3 appeared to effectively reduce the number of roots (Fig. 4). The distribution map of each haploblock in Japan showed some bias; as haploblock 1 was rarely identified in the Hokkaido region while haploblocks 2 and 3 appeared with a high frequency in the Hokkaido and Hokuriku regions, respectively (Fig. 5). Hokkaido is the northernmost region of Japan and is one of the northern limits for rice cultivation (Fujino *et al.* 2019). We assumed that haploblock 2 in the Hokkaido variety was derived from a landrace Akage (Supplemental Table 2), which is a cultivar that was used in early breeding programs at Hokkaido (Fujino *et al.* 2019). Several QTLs involved in heading date (Fujino *et al.* 2019, Yokoo *et al.* 1980) and cold tolerance (Saito *et al.* 2001, 2004) were used to help adapt rice to the Hokkaido environment. A cold tolerance QTL *Ctb1* was previously located at approximately 31.8 Mbp on chromosome 4 (Saito *et al.* 2004, Yonemaru *et al.* 2010), which was close to the location of *qNCR1* (32.7 Mbp on chromosome 4, Fig. 2). Therefore, we assumed that *qNCR1* was spread via the breeding program that focused on cold tolerance via linkage drag. The Hokuriku region is also where the Koshihikari cultivar was developed (Kobayashi *et al.* 2018). Since haploblock 3 was distributed only in a small number of cultivars including Koshihikari and Koshihikari-derived cultivars in Japan, we assumed that the cultivars with haploblock 3 have not been used after recent breeding using Koshihikari. We found that Morita-wase (landrace), an ancestor of Koshihikari (Kobayashi *et al.* 2018), harbored haploblock 3 (Supplemental Table 2). Therefore, we assumed that haploblock 3 in Morita-wase was inherited by Koshihikari and Koshihikari-derived cultivars.

The tiller number has a strong influence on root number (Kawakatsu *et al.* 2021). Because *qNCR1* was detected in juvenile seedlings with no tillering (Fig. 2A, 2B), we assumed that *qNCR1* did not influence the tiller number at juvenile stage. However, influence of *qNCR1* on tiller number at adult stage were not elucidated. Although we need further investigation to elucidate it before *qNCR1* will be used as breeding materials, these haploblocks of *qNCR1* could be potentially used to improve crown root number traits in rice. Koshihikari and Koshihikari-derived cultivars harbored haploblock 3, indicating that these cultivars could use haploblocks 1 and 2 to increase the root number. Almost all world-wide cultivars harbored haploblock 2 (Supplemental Table 1), indicating that these cultivars could use haploblocks 1 and 3 to increase and reduce the root number, respectively. The root number is associated with root density which is compatible with water and nutrient uptake (Marschner and Rengel 2012). Therefore, increasing the number of roots may improve the nutrient absorption characteristics of the crop. However, in some environments such as low water conditions, crops with reduced root numbers can perform better (Gao and Lynch 2016). Consequently, we propose that these haploblocks of *qNCR1* could be used to breed rice cultivars that are adapted to specific environments.

## Author Contribution Statement

ST designed the study, obtained the trait data at young growth period, analyzed all data, and wrote the manuscript. MY provided the SNP and indel data, supervised and conducted the GWAS. YU coordinated the project, designed the study, obtained the trait data at a middle growth period, and revised the manuscript.

## Supplementary Material

Supplemental Figures

Supplemental Tables

## Figures and Tables

**Table 1.** Candidate genes associated with root number

MSU ID	Gene	–Log_10_(*p*)	Description	Reference
*LOC_Os04g55140*		6.44	Retrotransposon protein, putative, Ty1-*copia* subclass, expressed	
*LOC_Os04g55030*	*OsCUL3b*	6.40	Cullin, putative, expressed	(Gingerich *et al.* 2005)
*LOC_Os04g55080*	*OsCNGC8*	6.40	Cyclic nucleotide-gated ion channel, putative, expressed	(Nawaz *et al.* 2014)
*LOC_Os04g55130*		6.40	Expressed protein	
*LOC_Os04g55350*		6.40	Retrotransposon protein, putative, Ty3-*gypsy* subclass, expressed	
*LOC_Os04g55450*		6.40	Transposon protein, putative, unclassified, expressed	
*LOC_Os04g55540*		6.40	Retrotransposon protein, putative, unclassified, expressed	
*LOC_Os04g55020*		5.40	Retrotransposon protein, putative, Ty3-*gypsy* subclass, expressed	
*LOC_Os04g55070*	*OsGA20ox8*	4.16	Gibberellin 20 oxidase 2, putative, expressed	(Ci *et al.* 2021)
