# Supplementary material for: Identification of a unique allele in the quantitative trait locus for crown root number in japonica rice from Japan using genome-wide association studies
Source: Breed Sci. 2022 Jul 1;72(3):222–31. doi: 10.1270/jsbbs.22010 (PMC9653191; doi:10.1270/jsbbs.22010)
Supplement: Supplementary file 1 — Supplemental Figures [file 72_222_s1.pdf]

doi: 10.1270/jsbbs.22010

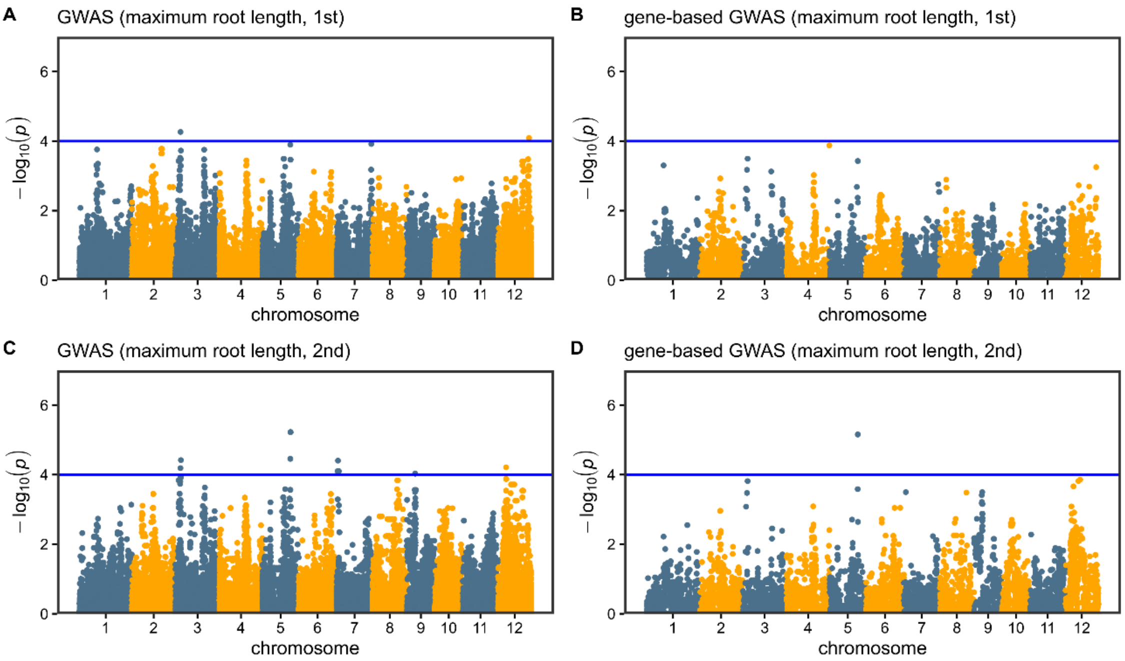

Supplemental Fig. 1. Manhattan plots for maximum root length. (**A**) GWAS and (**B**) gene-based GWAS results of the 1st trial. (**C**) GWAS and (**D**) gene-based GWAS results of the 2nd trial. The horizontal blue line indicates a threshold *p* value of 0.0001.

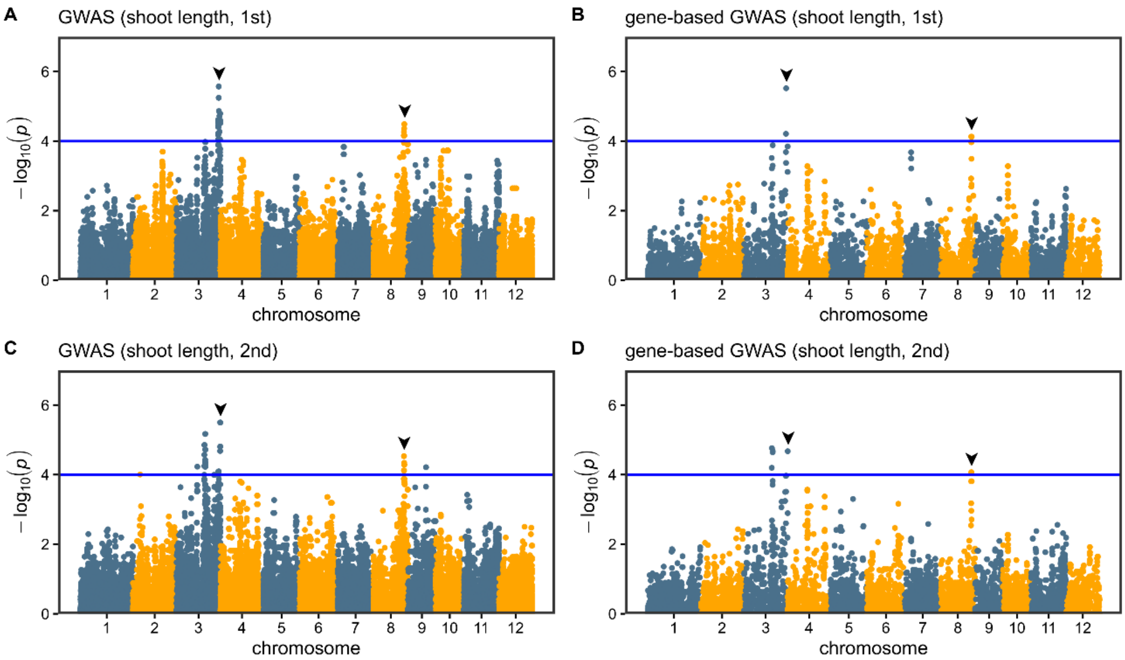

Supplemental Fig. 2. Manhattan plots for shoot length. (**A**) GWAS and (**B**) gene-based GWAS results of the 1st trial. (**C**) GWAS and (**D**) gene-based GWAS results of the 2nd trial. The horizontal blue line indicates a threshold *p* value of 0.0001. Arrowheads indicate the QTL commonly detected in **A**, **B**, **C**, and **D**.

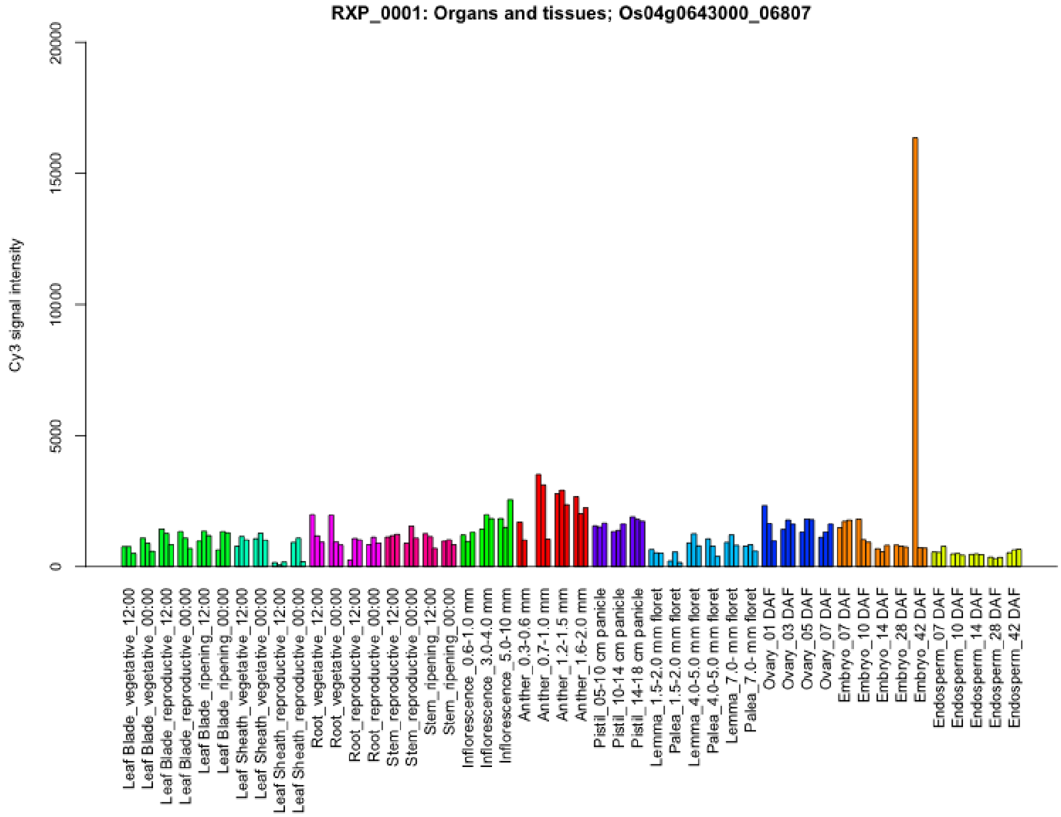

Supplemental Fig. 3. Expression profile of *LOC_Os04g55030*. This figure was downloaded from RiceXPro (https://ricexpro.dna.affrc.go.jp/).

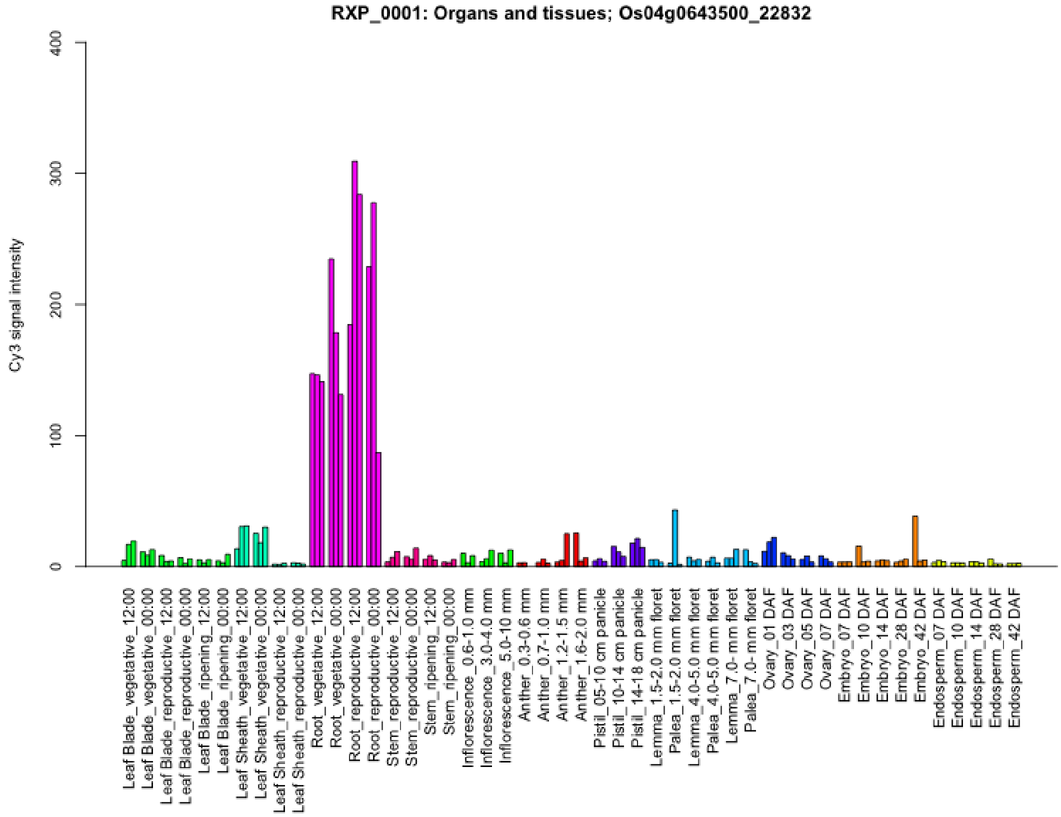

Supplemental Fig. 4. Expression profile of *LOC_Os04g55070*. This figure was downloaded from RiceXPro (https://ricexpro.dna.affrc.go.jp/).

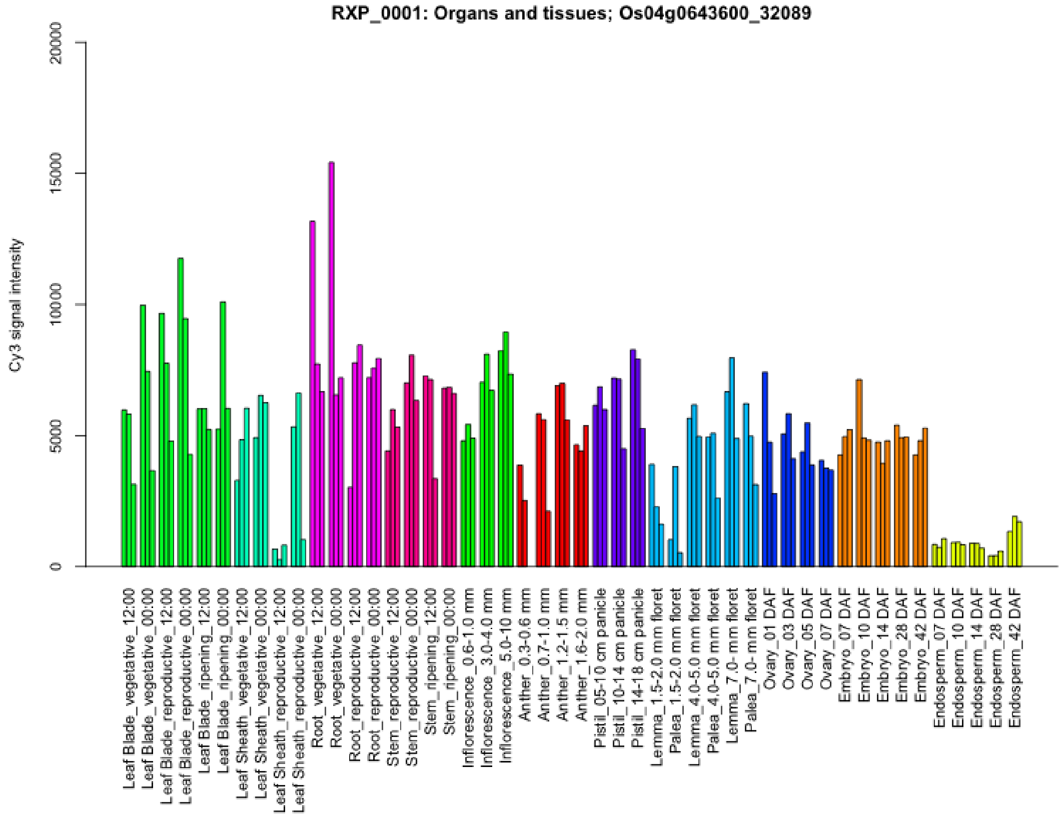

Supplemental Fig. 5. Expression profile of *LOC_Os04g55080*. This figure was downloaded from RiceXPro (https://ricexpro.dna.affrc.go.jp/).

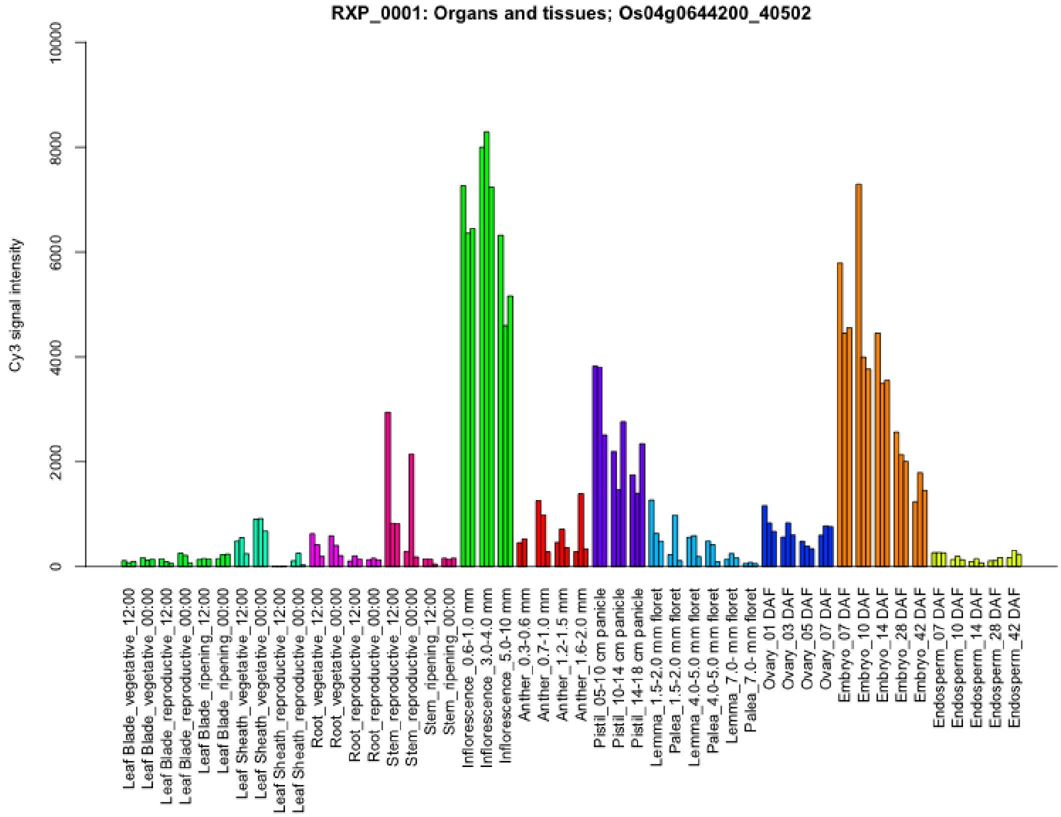

Supplemental Fig. 6. Expression profile of *LOC_Os04g55130*. This figure was downloaded from RiceXPro (https://ricexpro.dna.affrc.go.jp/).

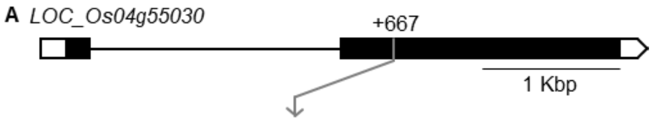

	codon (amino acid)	charge
Haplotype 1	GAA (E)	negative
Haplotype 2	AAA (K)	positive

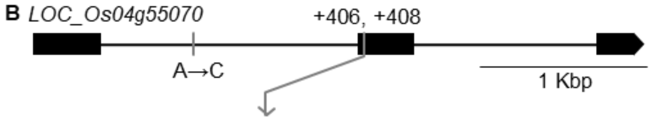

	codon (amino acid)	charge
Haplotype 1	TAT (Y)	neutral
Haplotype 2	GAG (E)	negative

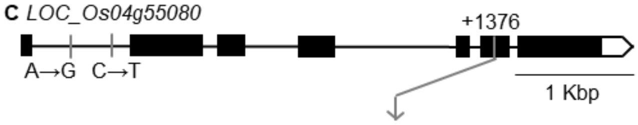

	codon (amino acid)	charge
Haplotype 1	TAT (Y)	neutral
Haplotype 2	TCT (S)	neutral

Supplemental Fig. 7. DNA mutations in the coding region of (**A**) *LOC_Os04g55030*, (**B**) *LOC_Os04g55070*, and (**C**) *LOC_Os04g55080*. The polygons and straight lines represent exons and introns, respectively. The colored polygon and white polygon the represent CDS (coding sequence) and UTR (untranslated region), respectively.

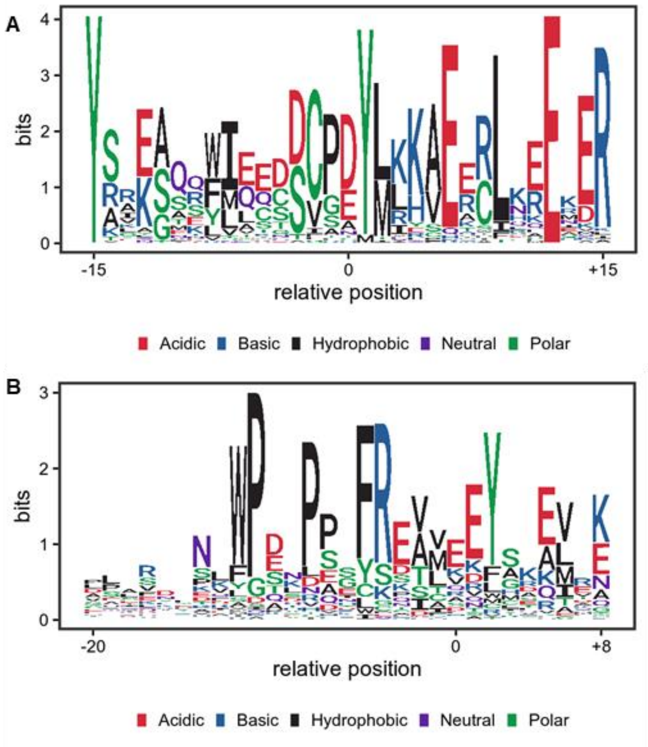

Supplemental Fig. 8. Amino acid sequence logo generated by the "ggseqlogo" R package. The relative position of the zero is the mutation position for (**A**) *LOC_Os04g55030* and (**B**) *LOC_Os04g55070*.

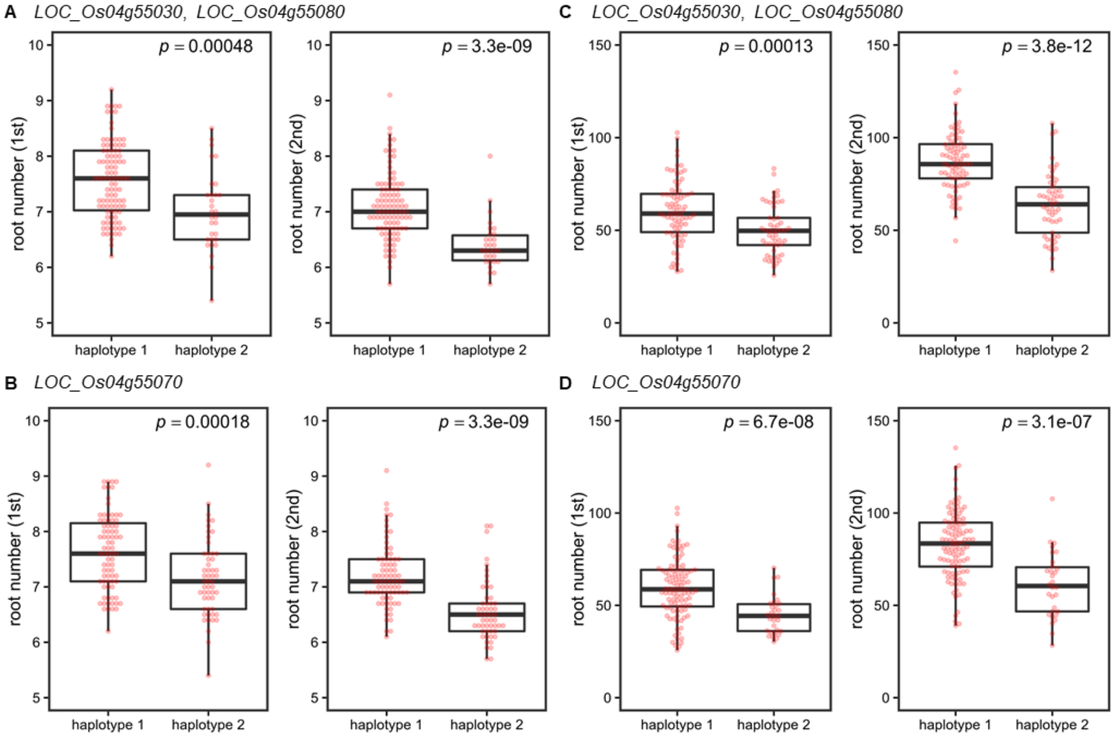

Supplemental Fig. 9. Boxplots indicating the root numbers in haplotypes 1 and 2 during the (**A**, **B**) early growth and (**C**, **D**) middle growth stages. The first and second trials are shown. The top and bottom of the boxes indicates the first and third quartiles, respectively. The center line represents the median, and the whiskers show the range of the observed values within 1.5 times the interquartile range from the hinges. The constituents of the box plot were marked as beeswarm points. Differences between the haplotypes were analyzed using Welch's *t*-test.
