# Supplementary material for: Identification of a unique allele in the quantitative trait locus for crown root number in japonica rice from Japan using genome-wide association studies
Source: Breed Sci. 2022 Jul 1;72(3):222–31. doi: 10.1270/jsbbs.22010 (PMC9653191; doi:10.1270/jsbbs.22010)
Supplement: Supplementary file 2 — Supplemental Tables [file 72_222_s2.pdf]

doi: 10.1270/jsbbs.22010

Supplemental Table 1. Haploblocks in the world rice core collection and the rice core collection of Japanese landraces.

Accession	Cultivar name	Haploblock	Accession	Cultivar name	Haploblock
WRC01	Nipponbare	1	WRC64	PadiKuning	2
WRC02	Kasalath	2	WRC65	Rambhog	2
WRC03	BeiKhe	2	WRC66	Bingala	2
WRC04	Jena035	2	WRC67	Phulba	2
WRC05	Naba	2	WRC68	KhaoNamJen	2
WRC06	PuluikArang	2	WRC97	ChinGalay	2
WRC07	Davao1	2	WRC98	Deejiaohualuo	2
WRC09	RyouSuisanKoumai	2	WRC99	HongCheuhZai	2
WRC10	QiuZaoZhong	2	WRC100	Vandaran	2
WRC11	Jinguoyin	2	JRC01	Gaisen_Mochi	2
WRC12	DaHongGu	2	JRC03	Hinode	2
WRC13	Asu	2	JRC04	Senshou	2
WRC14	IR58	2	JRC05	Yamada_Bake	2
WRC15	Co13	2	JRC06	Kaneko_B	1
WRC16	VaryFutsi	2	JRC07	Iruma_Nishiki	2
WRC17	Keiboba	2	JRC08	Okka_Modoshi	2
WRC18	Qingyu	2	JRC10	Hirayama	2
WRC19	DengPaoZhai	2	JRC11	Kahei	2
WRC20	Tadukan	2	JRC12	Oiran	2
WRC21	ShweNangGyi	2	JRC13	Bouzu_Mochi	1
WRC22	Calotoc	2	JRC14	Meguro_Mochi	1
WRC23	Lebed	2	JRC17	Akage	2
WRC24	Pinulupot1	2	JRC18	Hassokuho	2
WRC25	Muha	2	JRC19	Wataribune	2
WRC26	Jhona2	2	JRC20	Hosogara	2
WRC27	Nepal8	2	JRC21	Akamai_Kouchi	2
WRC28	Jarjan	2	JRC22	Mansaku	2
WRC29	KaloDhan	2	JRC23	Ishijiro	3
WRC30	AnjanaDhan	2	JRC24	Joushuu	1
WRC31	Shoni	2	JRC25	Dango	1
WRC32	Tupa121-3	2	JRC26	Aikoku	1
WRC33	Surjamukhi	2	JRC27	Ginbouzu	uncategorized
WRC34	ARC7291	2	JRC28	Shinriki_Mochi	1
WRC35	ARC5955	2	JRC29	Shichimenchou_Mochi	1
WRC36	Ratul	2	JRC30	Morita_Wase	3
WRC37	ARC7047	2	JRC31	Kameji	1
WRC38	ARC11094	2	JRC32	Omachi	1
WRC39	BadariDhan	2	JRC33	Shinriki	1
WRC40	Nepal555	2	JRC34	Kyoutoasahi	1
WRC41	Kaluheenati	2	JRC35	Kabashiko	1
WRC42	LocalBasmati	2	JRC36	Sekiyama	2
WRC43	Dianyu1	1	JRC37	Shinyamadaho_2	2
WRC44	Basilanon	2	JRC38	Nagoya_Shiro	2
WRC45	Masho	2	JRC39	Shiroine_Kemomi	1
WRC46	KhaoNok	2	JRC40	Akamai_Nagasaki	1
WRC47	Jaguary	1	JRC41	Akamai_Tokushima	2
WRC48	KhauMacKho	2	JRC42	Touboshi	2
WRC49	PadiPerak	2	JRC43	Akamai_Kantou	2
WRC50	Rexmont	2	JRC44	Karahoushi	2
WRC51	Urasan1	2	JRC45	Hiyadachitou	2
WRC52	KhauTanChiem	1	JRC46	Fukoku	1
WRC53	Tima	2	JRC47	Okabo	2
WRC55	Tupa729	2	JRC48	Hakamuri_Yokoyama	2
WRC57	Milyang23	2	JRC49	Rikutou_Rikuu_2	2
WRC58	NeangMenh	2	JRC50	Himenomochi	1
WRC59	NeangPhtong	2	JRC51	Shinshiuu	1
WRC60	Hakphaynhay	2	JRC52	Aichiasahi	1
WRC61	RadinGoiSesat	2	JRC53	Raiden	1
WRC62	Kemasin	2	JRC54	Houmanshinden_Ine	3
WRC63	Bleiyo	2			

Supplemental Table 2. Haploblocks of Japanese landraces used in GWAS.

Cultivar name	Origin	Haploblock
ASAHI (1)	Landrace	1
JIKKOKU	Landrace	1
AIKOKU	Landrace	1
ASAHI (2)	Landrace	1
OBA	Landrace	2
KAMEJI	Landrace	1
KAMENOO	Landrace	2
SHINRIKI	Landrace	1
TAKENARI	Landrace	1
AKAGE	Landrace	2
MORITA-WASE	Landrace	3
JYOSYU	Landrace	1
SENICHI	Landrace	2
GINBOUZU	Landrace	1
RIKUU 20	Landrace	1
KAMENOO 4	Landrace	2
OMACHI	Landrace	1
SEKITORI	Landrace	2
BOZU	Landrace	2
SHIROSENBON	Landrace	1
